# Digital Application of Clinical Staging to Support Stratification in Youth Mental Health Services: Validity and Reliability Study

**DOI:** 10.2196/45161

**Published:** 2023-09-08

**Authors:** Min K Chong, Ian B Hickie, Shane P Cross, Sarah McKenna, Mathew Varidel, William Capon, Tracey A Davenport, Haley M LaMonica, Vilas Sawrikar, Adam Guastella, Sharon L Naismith, Elizabeth M Scott, Frank Iorfino

**Affiliations:** 1 Brain and Mind Centre University of Sydney Camperdown Australia; 2 Orygen Parkville Australia; 3 Design and Strategy Division Australian Digital Health Agency Sydney Australia; 4 School of Health and Social Sciences University of Edinburgh Edinburgh United Kingdom; 5 Children's Hospital Westmead Clinical School Faculty of Medicine and Health The University of Sydney Sydney Australia; 6 Healthy Brain Ageing Program University of Sydney Sydney Australia; 7 St Vincent's and Mater Clinical School The University of Notre Dame Sydney Australia

**Keywords:** clinical staging, digital health solution, online diagnosis, service transformation, staged care, stratified care, youth mental health

## Abstract

**Background:**

As the demand for youth mental health care continues to rise, managing wait times and reducing treatment delays are key challenges to delivering timely and quality care. Clinical staging is a heuristic model for youth mental health that can stratify care allocation according to individuals’ risk of illness progression. The application of staging has been traditionally limited to trained clinicians yet leveraging digital technologies to apply clinical staging could increase the scalability and usability of this model in services.

**Objective:**

The aim of this study was to validate a digital algorithm to accurately differentiate young people at lower and higher risk of developing mental disorders.

**Methods:**

We conducted a study with a cohort comprising 131 young people, aged between 16 and 25 years, who presented to youth mental health services in Australia between November 2018 and March 2021. Expert psychiatrists independently assigned clinical stages (either stage 1a or stage 1b+), which were then compared to the digital algorithm’s allocation based on a multidimensional self-report questionnaire.

**Results:**

Of the 131 participants, the mean age was 20.3 (SD 2.4) years, and 72% (94/131) of them were female. Ninety-one percent of clinical stage ratings were concordant between the digital algorithm and the experts’ ratings, with a substantial interrater agreement (κ=0.67; *P*<.001). The algorithm demonstrated an accuracy of 91% (95% CI 86%-95%; *P*=.03), a sensitivity of 80%, a specificity of 93%, and an *F*_1_-score of 73%. Of the concordant ratings, 16 young people were allocated to stage 1a, while 103 were assigned to stage 1b+. Among the 12 discordant cases, the digital algorithm allocated a lower stage (stage 1a) to 8 participants compared to the experts. These individuals had significantly milder symptoms of depression (*P*<.001) and anxiety (*P*<.001) compared to those with concordant stage 1b+ ratings.

**Conclusions:**

This novel digital algorithm is sufficiently robust to be used as an adjunctive decision support tool to stratify care and assist with demand management in youth mental health services. This work could transform care pathways and expedite care allocation for those in the early stages of common anxiety and depressive disorders. Between 11% and 27% of young people seeking care may benefit from low-intensity, self-directed, or brief interventions. Findings from this study suggest the possibility of redirecting clinical capacity to focus on individuals in stage 1b+ for further assessment and intervention.

## Introduction

### Overview

Mental disorders present a substantial global challenge [1-3]. Poorly managed mental health too often leads to premature death [4] and has profound psychological, physical, and socioeconomic impacts. As 75% of mental disorders first emerge before the age of 25 years [5], early intervention and secondary prevention in young people are critical to halting the chronic impacts of mental illnesses [6,7].

Reforms in youth mental health services, digital platforms, and new models of care have been introduced in response to this critical need [8]. These models of care include stepped care, which offers low-intensity treatments for individuals’ specific needs [9], and integrated care, a multidisciplinary model of care that addresses diverse presentations of mental health in young people, including physical, mental, and functional concerns [10]. These innovations recognize that delayed access to treatment leads to increases in disengagement, discourages future help-seeking behavior, and results in poorer outcomes at higher costs [11-14]. However, despite such collective effort, demand for youth mental health services continues to surge, which impacts the delivery of timely and effective care [15-17]. Staged care is another model of care that aims to match the intervention intensity to the individual’s needs from the start of treatment [18]. A proposed solution for demand management in services is to use this stratification process (ie, staged care) to accurately and efficiently assess the complexity of a young person’s mental health condition and recommend appropriate care pathways [19].

### Clinical Staging Model

Clinical staging is a tool of risk stratification that guides decision-making for care pathways [20,21]. It stratifies help-seeking individuals based on illness severity, progression, and prognosis, ensuring that the intensity and timeliness of care match the urgency and complexity of their needs [18]. Current diagnostic systems, including the Diagnostic and Statistical Manual of Mental Disorders, Fifth Edition [22] and the International Classification of Diseases, Tenth Revision, Clinical Modification [23], are siloed and discrete, which limit their use in youth mental health for predicting an individual’s illness trajectory. On the contrary, the clinical staging model places an individual on a transdiagnostic continuum of their illness based on factors such as symptom duration, syndrome severity, and comorbidities. Hence, the clinical stage can indicate one’s clinical severity and risk of illness progression [21,24], facilitating clinicians to take preemptive action and allocate appropriate care from the start of treatment. This is especially useful for young people in the early stages of illness, as they often present with an admixture of subthreshold symptoms and comorbidities that do not fit into a specific diagnosis [3].

Furthermore, clinical staging assessments use a multidimensional framework that considers mental and physical health, social and occupational functioning, and substance use. Stratification systems that rely on single or state-based dimensions (ie, distress score or severity of symptoms) lack clinical usability as they fail to account for increased vulnerability associated with previous episodes of illness, comorbidity, and complex syndromes [25,26]. Studies show that while clinical predictors (eg, negative symptoms or psychotic characteristics) overlap across different mood disorders [27], nonclinical predictors (eg, family history or substance misuse) could strongly determine the emergence of specific mental illnesses in young people [26]. Therefore, this transdiagnostic model is a more appropriate risk stratification tool for early intervention in mental health services [18].

The clinical staging model provides a framework for differentiating individuals with persistent, full-threshold mental disorders (stage 2+), from those with nonspecific (stage 1a) and attenuated symptoms (stage 1b) [28,29]. A detailed set of criteria for clinical stages has been published elsewhere [20,30]; hence, only a short summary has been provided here. It is especially crucial to understand the distinction between stage 1a and stage 1b, as this can help predict the rate of illness progression [21]. While individuals in both stages 1a and 1b may present with subthreshold symptoms, they differ in clinical severity. Stage 1a individuals are characterized by nonspecific and mild symptoms of depression and anxiety disorders, whereas stage 1b individuals present a more complex symptom profile, including mild to severe symptoms of mood disorders, often with comorbidities. Therefore, while low-intensity services (eg, self-management and monitoring interventions) would be suitable for stage 1a, moderate-intensity services (eg, cognitive behavioral therapy) may be appropriate for stage 1b. Risk stratification using the clinical staging framework may help to prevent illness progression by aligning treatment intensity to an individual’s level of needs from the start of treatment.

This approach of the clinical staging model (ie, staged care) contrasts with the stepped care model, which initially assigns low-intensity care to all individuals and gradually elevates the level of care based on treatment response. Previous studies have demonstrated that staged care leads to faster and improved treatment outcomes compared to stepped care [31-33]. Additionally, it can facilitate early intervention and secondary prevention by detecting individuals who are susceptible to rapid illness progression. Therefore, incorporating clinical staging assessment at service intake can provide an opportunity to expedite care provision for those with complex needs and minimize their risk of illness progression.

### Digitalization of Clinical Staging

One of the main challenges of using clinical staging in services is the intense time and labor required for the assessment process. Conducting comprehensive multidimensional assessments can be time-consuming, and the associated costs and labor involved with educating clinicians pose major barriers to the widespread adoption of clinical staging. Relying solely on clinicians to perform these assessments would not be feasible for implementing clinical staging in mental health services at scale.

In recent years, digital technologies have been developed to improve access, efficiency, and quality in the health system. Technology-enabled assessment and care coordination have the potential to enhance access to standardized screening assessments and improve efficiency in the early detection of individuals with high and urgent needs [34-36]. Additionally, these technologies have the advantage of reaching a wider population, including services in rural and low-resource settings [37-39]. In the field of mental health, technology has been reported as a preferred method for self-disclosure and has been well-received among young people [40].

Hence, as a potential solution to the challenges of clinical staging implementation, a digital algorithm was developed to automatically assess clinical stage based on a multidimensional self-report questionnaire [41]. By leveraging this computational power, the algorithm offers a time- and cost-efficient solution for services to differentiate young people who require low-intensity interventions (stage 1a) from those who require moderate- to high-intensity interventions (stage 1b+) before their first consultation. This differentiation is an important early clinical decision for services to make with regard to service pathways and resource allocation. Validation of this digital algorithm may facilitate the implementation of clinical staging at scale to improve service efficiencies and expedite care pathways.

Therefore, the aim of this study was to assess the validity of the digital algorithm to accurately allocate young people to stage 1a or stage 1b+, compared to expert raters. Demographic and clinical characteristics of individuals in each clinical stage were compared to further evaluate the algorithm.

## Methods

### Participants

A total of 131 participants aged between 16 and 25 years were randomly selected from help-seeking young people who presented at primary mental health care services (*headspace* [10]). All young people were deemed eligible if they presented for the first time to a participating *headspace* service between November 2018 and March 2021 and used the Innowell Platform to provide assessment information. The participating *headspace* services were in Central and Eastern Sydney, Queensland, and South Australia. The cohort was a subset of the total presentation at these services, representing the clinical characteristics of the help-seeking young people.

### Innowell Platform

Innowell Platform is a digital technology that supports individuals’ mental health and well-being by facilitating personalized and measurement-based care [41]. It is a web-based platform that provides multidimensional assessments and immediately generates outcome reports that can be used to identify individuals’ psychological, functional, and physical needs [42]. Individuals can review their clinical assessment scores with their health professionals on their personalized dashboard, and their symptom progression can be tracked by repeating the questionnaires over time. The platform offers other resources, such as links to fact sheets, apps, or web-based tools, according to individuals’ clinical needs.

### Procedures

Registration on the Innowell Platform was part of the intake process at participating mental health clinics. All participants completed a self-report questionnaire on the Innowell Platform before their initial consultation with a clinician. The questionnaire assessed individuals’ background information, including demographics, current education and employment participation, mental health concerns (ie, psychological distress, depressed mood, anxiety, mania-like experiences, psychosis-like experiences, and posttraumatic experiences), self-harm and suicidal behaviors, tobacco, alcohol and other substance use, physical activity, sleep-wake cycles, mental health history, eating behaviors, and social connectedness. After the web-based assessment, the digital algorithm embedded in the platform automatically allocated individuals to stage 1a or stage 1b+ based on the questionnaire results. Details of the algorithm have been published previously [41]. Only 2 stages were allocated (stage 1a or 1b+), as individuals in clinical stage 1b or above should be directed to further clinical assessment to examine the level of care they require.

### Measures

For the purposes of this study, the following measures were specifically selected and included for analysis: demographics, mental health, suicidality, functioning, alcohol and substance use, eating behavior, and circadian disturbance.

#### Demographics

Participant age, gender, highest level of education, and current education, employment, and training status (used to determine “not in education, employment, or training” [NEET] status) were collected.

#### Mental Health

Current psychological distress was assessed using the Kessler Psychological Distress Scale (K10) [43], a well-validated and widely used measure of general psychological symptoms and distress in adult and adolescent populations in both clinical and community settings. Manic-like experiences over the last 12 months were assessed using a screener derived from the Altman Self-Rating Scale [44]. To assess subclinical psychotic symptoms, the Prodromal Questionnaire (PQ-16) was used [45]. The Overall Anxiety Severity and Impairment Scale (OASIS) [46] and Quick Inventory of Depressive Symptomatology (QIDS) [47] were used to assess anxiety and depressed mood, respectively. Participants were also asked, “Have you ever experienced a major mental health or behavioral problem that has affected your everyday life?” to determine any mental health problems or hospitalization history.

#### Suicidality

The Suicide Ideation Attributes Scale (SIDAS) was used to assess suicidal ideation over the past month. The scale is comprised of 5 items that assess the frequency of suicidal thoughts and attempts, related distress, and impact on daily activities on a 10-point Likert scale. A score above 21 indicated a high risk of suicidal behavior. The scale is a valid web-based measure with strong internal reliability (Cronbach α=.91) [48]. A self-harm history question was adapted from the Brief Non-Suicidal Self-Injury Assessment Tool [49], and a suicide attempt history question was extracted from the Columbia-suicide severity rating scale [50].

#### Functioning

Everyday functioning was assessed using the Work and Social Adjustment Scale [51]. A total of 5 items were scored using an 8-point Likert scale. Participants were asked to rate their level of agreement with statements such as “Because of my mental health, my ability to work is impaired.” Scores 0 and 8 represented “not at all” and “very severely,” respectively.

#### Alcohol and Substance Use

Participants’ alcohol use, onset age, and related impairments were assessed using a combination of 3 measures. The frequency and impact of alcohol use were assessed with questions extracted from the Alcohol Use Disorders Identification Test [52] and the Alcohol, Smoking, and Substance Involvement Screening Test [53], respectively. One question on age onset was added based on past literatures [54-56]. Additionally, frequency of cannabis use and its associated impacts on health, social, legal, or financial domains were assessed using questions extracted from the Alcohol, Smoking, and Substance Involvement Screening Test [53].

#### Eating Behavior

Questions were adapted from the investigator-based interview, the Eating Disorders Examination (adapted version) [57]. The questions assessed behaviors such as binge eating, purging, strict dieting, and body image importance. Body image importance was rated on a 6-point Likert scale. A score of 0 indicated “not at all important,” and score 6 indicated “most important.” Individuals were rated as having an abnormal eating behavior if they experienced binge eating, purging, and diet restriction over the past 3 months and gave their body image importance a rating above 3.

#### Circadian Disturbance

Individuals’ sleep and wake time, sleep duration, and quality of sleep were assessed using questions from the Pittsburgh Sleep Quality Index [58] and the Munich Chronotype Questionnaire [59]. An additional question on restorative sleep was included based on its significance in the past literature [60].

### Clinical Staging Assessment

To evaluate the validity of the digital algorithm, expert psychiatrists (IBH and EMS), who were involved with the development of the clinical stage model [20], allocated clinical stages using the results of the web-based questionnaire. An expert rating represents the collaborative clinical stage allocation by the 2 psychiatrists, which represents the current gold standard and best practice application of clinical staging [20]. The expert rating was conducted independently to the digital algorithm, and all participants were allocated to either stage 1a or 1b+.

### Statistical Analyses

All statistical analyses were performed using the RStudio software (version 4.2.1; R Foundation) [61]. Demographic, functioning, and clinical characteristics between stages 1a and 1b+ were compared using the nonparametric Mann-Whitney *U* test for continuous variables and the chi-square test for categorical variables. To further evaluate discrepancies between the expert and the digital algorithm assigned clinical stages, pairwise comparisons were conducted with concordant and discordant ratings. Due to the number of comparisons made within the dataset, a Bonferroni correction was used. The α value of .05 was adjusted to *P*<.001. Cases with missing measures were excluded from the analyses.

The reliability of the digital algorithm was assessed using the Cohen κ statistics [62] and the confusion matrix [63]. A κ coefficient between 0.01-0.20 was interpreted to be in slight agreement; κ=0.21-0.40 in fair agreement; κ=0.41-0.60 in moderate agreement; κ=0.61-0.80 in substantial agreement; and κ=0.81-0.99 in almost perfect agreement [64]. Using the confusion matrix, accuracy, positive predictive value (precision), sensitivity (recall), and the *F*_1_-score were analyzed. Accuracy represents the percentage of correct ratings in the total sample, and the *F*_1_-score presents a balanced mean using precision and recall. A higher *F*_1_-score indicates a greater rate of both precision and recall.

### Ethics Approval

The Northern Sydney Local Health District Human Research Ethics Committees approved this study (HREC/17/HAWKE/480), and all participants gave web-based informed consent (through an opt-out process).

## Results

### Sample Characteristics

A total of 131 young people were included in the analyses (Table 1). The mean age of participants was 20.3 (SD 2.4) years, 72% (94/131) of them were female, and 36% (47/131) of them completed tertiary education. Most participants reported that they did not have a disability (91%, 115/126).

**Table 1:** Differences in demographic and clinical characteristics among participants with concordant and discordant stage ratings.

Characteristics		Total participant (n=131)	Stage 1a (agree)^a^ (n=16)	Stage 1b+ (agree)^b^ (n=103)	Stage 1b+ (disagree)^c^ (n=8)	Comparison ^d^	
						Stage 1a (agree) versus stage 1b+ (disagree)	Stage 1b+ (agree) versus stage 1b+ (disagree)
Age (years), mean (SD)		20.3 (2.4)	20.6 (2.5)	20.1 (2.5)	21.5 (1.9)	— ^e^	—
Female, n (%)		94 (71.8)	11 (68.8)	75 (72.8)	4 (50)	—	—
**Education, n (%)**							
	Secondary	84 (64.1)	4 (25)	75 (72.8)	4 (50)	—	—
	Tertiary	47 (35.9)	12 (75)	28 (27.2)	4 (50)	—	—
Mental health history, n (%)^f^		101 (78.3)	8 (50)	86 (84.3)	3 (42.9)	—	—
With disability, n (%)^f^		11 (8.7)	0 (0)	10 (10.1)	1 (12.5)	—	—
Functioning, mean (SD)		20 (9.1)	8.9 (5.1)	21.9 (8.6)	17.4 (3)	<.001	
**Clinical presentation**							
	Psychological distress, mean (SD)	32.8 (8.6)	22.2 (6.4)	35.1 (7.6)	25.8 (4.8)	—	—
	Depression, mean (SD)^f^	14.8 (5.7)	6.8 (3.5)	16.5 (4.9)	9.9 (3.5)	—	<.001
	Anxiety, mean (SD)^f^	10.1 (5.0)	5.1 (3.4)	11.1 (4.7)	5.6 (1.1)	—	<.001
	Manic-like experiences, n (%)^f^	37 (40.7)	1 (12.5)	36 (48.6)	0 (0)	—	—
	Psychotic-like experiences, n (%)^f^	58 (45.7)	0 (0)	58 (57.4)	0 (0)	—	—
	Circadian disturbance, n (%)^f^	86 (67.2)	5 (31.3)	73 (72.3)	5 (71.4)	—	—
	Abnormal eating behavior, n (%)^f^	9 (7.2)	0 (0)	9 (9.2)	0 (0)	—	—
**Self-harm and suicidal thoughts and behaviors, n (%)**							
	Self-harm history^f^	72 (55.4)	3 (18.8)	66 (64.1)	2 (28.6)	—	—
	Suicidal ideation, mean (SD)	9.8 (12.6)	1.7 (4)	12.1 (13.2)	1.4 (2.7)	—	—
	Suicide attempt history^f^	49 (37.7)	0 (0)	49 (47.6)	0 (0)	—	—
**Alcohol and other substance misuse, mean (SD)**							
	Alcohol use^f^	4 (3.1)	2.9 (2.8)	4.1 (3.1)	6.1 (2.1)	—	—
	Cannabis use^f^	2.1 (3.4)	0.5 (0.9)	2.3 (3.6)	2.9 (3.8)	—	—

^a^ Stage 1a by the algorithm and experts.

^b^ Stage 1b+ by the algorithm and experts.

^c^ Rated stage 1b+ by experts and stage 1a by the algorithm.

^d^*P*<.001 for statistical significance

^e^Not available.

^f^There were some missing data from the sample for following demographic characteristics. Mental health history, n=129; with disability, n=126; depression, n=126; anxiety, n=129; manic-like experiences, n=91; psychotic-like experiences, n=127; circadian disturbance, n=128; abnormal eating behavior, n=125; self-harm history, n=130; suicide attempt history, n=130; alcohol use, n=124; cannabis use, n=126. Percentages have been calculated only with available data to represent the proportion of young people with corresponding characteristics. Corresponding measures; Functioning, Work and Social Adjustment Scale; Psychological distress, Kessler-10; Depression, Quick Inventory of Depressive Symptomatology; Anxiety, Overall Anxiety Severity and Impairment Scale; Manic-like experiences, Altman Self-Rating Mania Scale; Psychotic-like experiences, Prodromal Questionnaire; Circadian disturbances, Pittsburgh Sleep Quality Index and Munich Chronotype Questionnaire; Abnormal eating behavior, Eating Disorder Examination (adapted version); Self-harm history, Brief Non-Suicidal Self-Injury Assessment Tool; Suicidal ideation, Suicide Ideation Attributes Scale; Suicide attempt history, Columbia-Suicide Severity Rating Scale; Alcohol use, Alcohol Use Disorders Identification Test; Cannabis use, Alcohol, Smoking and Substance Involvement Screening Test.

Overall, the sample reported moderate to severe mental health concerns. The sample displayed severe psychological distress (K10 total mean 32.8, SD 8.6; range 10-50), moderately depressed mood (QIDS total mean 14.8, SD 5.7; range 0-27), and moderate anxiety symptoms (OASIS total mean 10.1, SD 5.0; range 0-20). Manic-like experiences (37/91, 41%) and psychotic-like experiences (58/127, 46%) were commonly reported. Over half of the participants had a history of self-harm (72/130, 55%), 38% (49/130) with previous suicide attempts and one-fifth exhibited high suicidality (26/131, 20%). A large proportion reported sleep disturbances (86/125, 69%).

### Interrater Reliability

Out of the 131 participants, 119 (91%) clinical stages allocated by the digital algorithm were concordant with those assigned by experts (Table 2). Among the 12 discordant ratings, the algorithm assigned 8 participants to a less severe clinical stage (ie, stage 1a) compared to the experts. Cohen κ of 0.67 indicated a substantial agreement between the algorithm and the expert ratings, and the digital algorithm achieved an accuracy of 91% (95% CI 85%-95%; *P*=.03) and an *F*_1_-score of 73%. The sensitivity and specificity of the algorithm were 80% and 93%, respectively.

**Table 2:** Confusion matrix comparing clinical stage assessments between digital algorithm and expert rating.

Expert rating	Digital algorithm	
	Stage 1a (n=24), n (%)	Stage 1b+ (n=107), n (%)
Stage 1a (n=20)	16 (12)	4 (3)
Stage 1b+ (n=111)	8 (6)	103 (79)

### Demographic, Functional, and Clinical Characteristics Differences Between Concordant and Discordant Ratings

To further develop the algorithm, the demographic, functional, and clinical differences between concordant and discordant cases were evaluated. The first set of analyses compared participants with concordant stage 1a with those who were allocated to stage 1a by algorithm but to stage 1b+ by the experts (addressed as discordant stage 1b+ hereafter). The analyses showed a significant functional impairment in the discordant stage 1b+ group compared to concordant stage 1a group (*U*=7.5; *z*=–3.3; *P*<.001; Table 1).

Then, the second set of analyses compared participants with concordant stage 1b+ and the discordant stage 1b+ group. The results showed that the discordant stage 1b+ group had less symptoms of depression (*U*=77; *z*=–3.2; *P*<.001) and anxiety (*U*=86; *z*=–3.2; *P*<.001; Table 1).

Due to the small sample size, no analysis was conducted for participants with discordant stage 1a rating (allocated to stage 1a by the experts and stage 1b+ by the algorithm).

### Demographic, Functional, and Clinical Characteristics of Stage 1a and Stage 1b+

To evaluate the characteristics that differentiated stage 1a and stage 1b+ for each examiner, participants were grouped by stages per examiner, and pairwise comparison analyses of their characteristics were performed. For both examiners, participants in stage 1b+ had greater psychological distress (algorithm: *U*=305.5, *z*=–5.7, *P*<.001; expert: *U*=349, *z*=–4.7, *P*<.001), depressed mood (algorithm: *U*=192, *z*=–6.2, *P*<.001; expert: *U*=252, *z*=–5.3, *P*<.001), anxiety symptoms (algorithm: *U*=343.5, *z*=–5.3, *P*<.001; expert: *U*=527.5, *z*=–3.5, *P*<.001), and functional impairment (algorithm: *U*=447.5, *z*=–4.8, *P*<.001; expert: *U*=379.5, *z*=–4.5, *P*<.001). Further, psychotic-like experiences were more common in stage 1b+ (algorithm: *χ*^2^_1_=20.2, *P*<.001; expert: *χ*^2^_1_=17.8, *P*<.001). However, episodes of self-harm (*χ*^2^_1_=11.2, *P*<.001) and mental illness history (*χ*^2^_1_=13.2, *P*<.001) were only significantly different between the algorithm-rated stage 1a and stage 1b+ participants. Additionally, the level of education (*χ*^2^_1_=13.8, *P*<.001) was only different between the expert-rated stage 1a and stage 1b+ participants (Multimedia Appendix 1).

## Discussion

### Principal Findings

This study demonstrates that a digital algorithm [41] can differentiate individuals in very early stages of mental illness (stage 1a) from those with increased risk of illness progression or more developed syndromes (stage 1b+) based on a web-based multidimensional self-report assessment (accuracy 91%; κ=0.67). Validation of this tool provides support for its further evaluation and use in services for stratification, which may help youth mental health services to reduce unnecessary delays for assessment and treatment, as well as enhance the quality of care.

### Evaluation of the Digital Algorithm

Our results show that the algorithm was more conservative when assessing clinical stage, indicating that it had a greater tendency to assign lower and less severe clinical stage (ie, stage 1a) compared to the experts (Table 2). Participants who were rated higher by the experts (stage 1b+) than the algorithm (stage 1a) displayed greater functional impairment, but lower levels of depression and anxiety symptoms. This indicates that while the algorithm can detect symptom severity (eg, depression and anxiety symptoms), it may not be as sensitive to other multidimensional factors (eg, functioning) that influence the risk of illness progression [21]. However, the conservativeness of the digital algorithm aligns with the recommended practice of allocating lower stages based on uncertainty [20]. While, in practice, all digitally allocated clinical stages should be reviewed by clinicians, the presented digital algorithm has demonstrated the capability to differentiate young people in the early stages of illness from those at later stages.

### Clinical Usability of the Clinical Staging Algorithm in Mental Health Services

**Figure 1:**
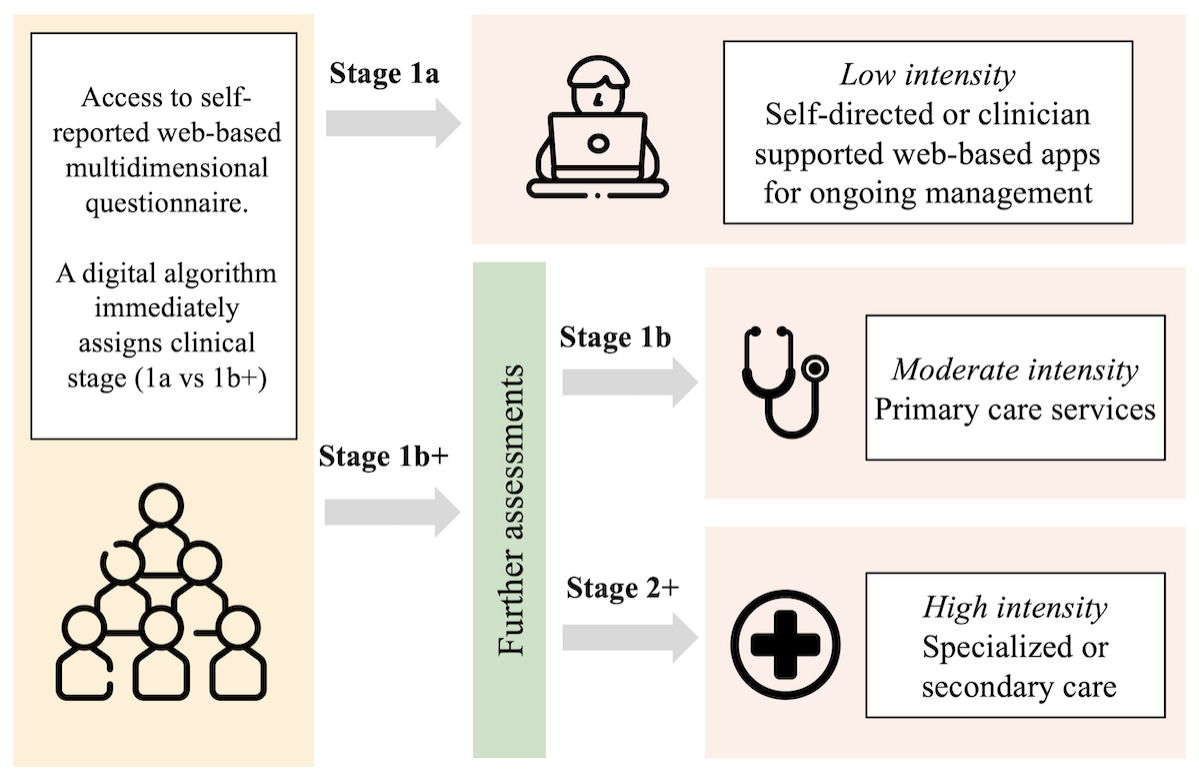
Care pathway transformation using the digital algorithm.

When used in mental health services, the digital algorithm validated here could promote standardization and implementation of clinical staging at scale. Traditionally, clinical staging assessments require an intensive assessment by a clinician, which takes significant time and resources. For this reason, the heuristic has limited use, particularly when considering the large demand for services. The digitalization of clinical staging proposes a potential solution to this problem by condensing a large volume of biopsychosocial measures in a self-reported assessment into a clinical stage, which can then be translated into actionable treatment strategies. The major usability of this digital algorithm focuses on the differentiation of stage 1a from stage 1b+. Young people in stage 1b and stage 2+ may be similar across many clinical characteristics, making it difficult to differentiate without further assessment. However, most stage 1a cases could readily be distinguished from the population. The large degree of concordance here supports this hypothesis and illustrates how the digital algorithm could be used to direct young people to the appropriate level of care. Young people in stage 1a could be directed to web-based, self-directed, or brief clinician-supported resources (eg, web-based CBT or psychoeducation [65-67]), while using digital technologies to track their symptoms and monitor any changes (Figure 1 [68,69]). Concurrently, access to early intervention and further assessments can be expedited for individuals with attenuated or more developed syndromes (stage 1b+) so that a decision can be made about the type and intensity of care required for these individuals, who are more likely to have complex presentations.

The proportion of stage 1a represented in this cohort was smaller than previous reports on clinical stage presentation at youth mental health services with larger sample sizes [70-72]. The lowest estimate is 15% (the current sample), but other independent samples suggest that the proportion could be as high as 30% [21,70,71]. This means that based on the digital algorithm’s accuracy (91%) and *F*_1_-score (73%), between 11% and 27% of total presentations to youth mental health services can accurately be allocated to stage 1a. This could prove to be a beneficial tool for services to manage demand and stratify young people to the appropriate level of care. Directing young people at stage 1a to low-intensity interventions may reduce unnecessary delays in accessing evidence-based treatments that are most likely to be effective for their stage of illness. This may then facilitate access to early interventions for those young people with an increased risk of mental illness progression.

### Limitations

There are several limitations to the current study. First, this study only assessed the interrater reliability between the digital algorithm and 2 expert psychiatrists’ clinical stages. A team-based assessment of clinical stage should be the focus of future studies to provide stronger validation of the digital algorithm [73]. Second, the sample may not be representative of the general population presenting to youth mental health services. For example, the age difference between stage 1a and stage 1b+, which has previously been reported [18], was not reflected in this study. Future studies would benefit from validating the algorithm in other representative samples. However, the impact of representativeness on the analyses reported here is minimal since the algorithm is rule-based and expert assessors were drawing upon their own experience. Lastly, the digital algorithm was validated by assessing concordance with experts who used the same questionnaire and assessment results. The chosen methodology aims to focus on comparing algorithm performance against the information available to clinicians who are assessing the needs of young people before a face-to-face assessment. Therefore, it was important for the algorithm to align with clinical judgment based on the same questionnaire. Future validation should focus on comparing performance to a separate clinical interview, which would provide support for its wider clinical usability in services.

### Conclusions

This study validates a digital algorithm for clinical staging. We present a digital health solution for managing demand in current youth mental health services by applying clinical staging to allocate care according to an individual’s risk of illness progression. This work provides preliminary evidence for the use of the digital algorithm as a stratification tool for efficient treatment allocation. There are many avenues for future research to further the development and evaluation of this algorithm, which includes assessing the longitudinal outcomes of young people stratified into each group and identifying its impact on treatment outcomes and waitlist management in youth mental health settings.

## Appendix

Multimedia Appendix 1 Differences in demographic, clinical, and functional characteristics between clinical stages assessed by experts and the digital algorithm.

## Abbreviations

K10: Kessler Psychological Distress Scale
NEET: not in education, employment, or training
OASIS: Overall Anxiety Severity And Impairment Scale
PQ-16: Prodromal Questionnaire-16
QIDS: Quick Inventory Of Depressive Symptomatology-Self-Report
SIDAS: Suicide Ideation Attributes Scale

## Data Availability

The data presented in this manuscript are available upon request.
