# Supplementary material for: Digital Application of Clinical Staging to Support Stratification in Youth Mental Health Services: Validity and Reliability Study
Source: JMIR Form Res. 2023 Sep 8;7:e45161. doi: 10.2196/45161 (PMC10517388; doi:10.2196/45161)
Supplement: Multimedia Appendix 1 [file formative_v7i1e45161_app1.docx]

**Supplementary Table 1**: Differences in demographic, clinical, and functional characteristics between clinical stages assessed by experts and the digital algorithm.

| **Characteristics** | **N (%)** | | |  | | |  | |  |
| --- | --- | --- | --- | --- | --- | --- | --- | --- | --- |
|  | **Expert** | | | | **Digital algorithm** | | **Comparison^e^** | |  |
|  | Stage 1a^a^ | Stage 1b+^b^ | | | Stage 1a^c^ | Stage 1b+^d^ | a v b | c v d |  |
| Number of participants | 20 | 111 | | | 24 | 107 |  |  |  |
| Age (years), mean (SD) | 20.5 (2.3) | 20.2 (2.5) | | | 20.9 (2.3) | 20.1 (2.5) |  |  |  |
| Female | 15 (75.0) | 79 (71.2) | | | 15 (62.5) | 79 (73.8) |  |  |  |
| Education |  |  | | |  |  | <.001 |  |  |
| Secondary | 5 (25) | 79 (71.2) | | | 8 (33.3) | 76 (71) |  |  |  |
| Tertiary | 15 (75) | 32 (28.8) | | | 16 (66.7) | 31 (29) |  |  |  |
| Mental health history^f^ | 12 (60) | 89 (81.7) | | | 11 (47.8) | 90 (84.9) |  | <.001 |  |
| Has disability^f^ | 0 (0) | 11 (10.3) | | | 1 (4.3) | 10 (9.7) |  |  |  |
| Functioning, mean (SD) | 11.3 (7.8) | 21.6 (8.4) | | | 11.8 (6) | 21.9 (8.6) | <.001 | <.001 |  |
| Clinical presentation |  |  | | |  |  |  |  |  |
| Psychological distress, mean (SD) | 23.9 (7.1) | 34.4 (7.8) | | | 23.4 (6.1) | 34.9 (7.6) | <.001 | <.001 |  |
| Depression, mean (SD) ^f^ | 8.1 (4.3) | 16 (5.1) | | | 7.7 (3.7) | 16.4 (4.8) | <.001 | <.001 |  |
| Anxiety, mean (SD) ^f^ | 6.4 (4.8) | 10.8 (4.7) | | | 5.2 (2.8) | 11.2 (4.7) | <.001 | <.001 |  |
| Manic-like experiences^f^ | 1 (9.1) | 36 (45.0) | | | 1 (7.1) | 36 (46.8) |  |  |  |
| Psychotic-like experiences^f^ | 0 (0) | 58 (54.2) | | | 0 (0) | 58 (55.2) | <.001 | <.001 |  |
| Circadian disturbance^f^ | 8 (40) | 78 (72.2) | | | 10 (43.5) | 76 (72.4) |  |  |  |
| Abnormal eating behavior^f^ | 0 (0) | 9 (8.6) | | | 0 (0) | 9 (8.8) |  |  |  |
| Self-harm and suicidal thoughts and behaviors | | |  |  |  |  |  |  |  |
| Self-harm history^f^ | 4 (20) | 68 (61.8) | | | 5 (21.7) | 67 (62.6) |  | <.001 |  |
| Suicidal ideation, mean (SD) | 1.4 (3.6) | 11.4 (13.1) | | | 1.6 (3.5) | 11.7 (13.2) |  |  |  |
| Suicide attempt history^f^ | 0 (0) | 49 (44.5) | | | 0 (0) | 49 (45.8) |  |  |  |
| Alcohol and other substance misuse, mean (SD) | | | | | | | | | |
| Alcohol use^f^ | 2.7 (2.6) | 4.2 (3.1) | | | 3.9 (3.0) | 4.0 (3.1) |  |  |  |
| Cannabis use^f^ | 0.7 (1.5) | 2.4 (3.6) | | | 1.2 (2.4) | 2.3 (3.6) |  |  |  |

^a^ Participants who were allocated with stage 1a by the experts

^b^ Participants who were allocated with stage 1b+ by the experts

^c^ Participants who were allocated with stage 1a by the digital algorithm

^d^ Participants who were allocated with stage 1b+ by the digital algorithm

^e^ *P*<.001 for statistical significance

^f^Measures with missing data was excluded in the analyses.

Corresponding measures: Functioning, Work and Social Adjustment Scale; Psychological distress, Kessler-10; Depression, Quick Inventory of Depressive Symptomatology; Anxiety, Overall Anxiety Severity and Impairment Scale; Manic-like experiences, Altman Self-Rating Mania Scale; Psychotic-like experiences, Prodromal Questionnaire; Circadian disturbances, Pittsburgh Sleep Quality Index and Munich Chronotype Questionnaire; Abnormal eating behavior, Eating Disorder Examination (adapted version); Self-harm history, Brief Non-Suicidal Self-Injury Assessment Tool; Suicidal ideation, Suicide Ideation Attributes Scale; Suicide attempt history, Columbia-Suicide Severity Rating Scale; Alcohol use, Alcohol Use Disorders Identification Test; Cannabis use, Alcohol, Smoking and Substance Involvement Screening Test. Abbreviations: SD, standard deviation; v, versus.
